# Examining the Potential Role of Opioid Settlement Funds in the Face of Impending Federal Budget Reductions for Substance Use Disorders

**DOI:** 10.1111/1475-6773.70094

**Published:** 2026-02-24

**Authors:** Zoe Lindenfeld, Amanda I. Mauri, Sachini Bandara, Jonathan H. Cantor, Ryan K. McBain, Abigail Winiker

**Affiliations:** ^1^ Edward J. Bloustein School of Planning and Public Policy Rutgers University New Brunswick New Jersey USA; ^2^ Department of Health Policy and Management, School of Public Health University of Maryland College Park Maryland USA; ^3^ Department of Mental Health Johns Hopkins Bloomberg School of Public Health Baltimore Maryland USA; ^4^ Department of Health Policy and Management Johns Hopkins Bloomberg School of Public Health Baltimore Maryland USA; ^5^ RAND Santa Monica California USA; ^6^ RAND Arlington Virginia USA

**Keywords:** addiction, health policy, opioids, substance use disorder

## Abstract

**Objective:**

To assess the potential of opioid settlement dollars disbursed to state and local governments to replace or supplement federal substance use disorder (SUD) funding.

**Study Setting and Design:**

For each state, we calculated the percentage of Substance Use and Mental Health Services Administration (SAMHSA) SUD funding for each state that can be offset by settlement funds.

**Data Sources and Analytic Sample:**

We estimated annual opioid settlement distributions (2022–2038) from KFF Health News and SAMHSA‐funded SUD awards (2024) from SAMHSA's website and the Tracking Accountability in Government Grants System.

**Principal Findings:**

Across states, the mean difference between SAMHSA SUD funds and settlement funds was $56.83 (SD: $53.76) per capita, and the mean percentage of SAMHSA SUD funding that could be replaced by settlement funds was 51.16 (SD: 28.46) per capita. Oregon was the only state where settlement disbursements exceeded SAMHSA SUD funding.

**Conclusions:**

Substantial gaps remain between current federal SUD funding and what opioid settlement funds can offset. Our findings underscore that opioid settlement funds are not a viable replacement for federal funding, both because they were never intended to serve this purpose, and because they are insufficient in scale.

**Callout Box**

- What is known about this topic?
  ○ The Substance Abuse and Mental Health Services Administration (SAMHSA) has been one of the largest sources of federal funding for substance use disorder (SUD) treatment and prevention services.
  ○ However, federal funding for SUD treatment and prevention services is at risk, with SAMHSA facing substantial budgetary reductions as of 2025.
  ○ As federal support potentially recedes, some states are turning to opioid settlement funds to sustain financial support for SUD treatment and prevention services.
- What this study found
  ○ We compare SAMHSA federal funding awards with projected state‐level opioid settlement disbursements to evaluate the extent to which settlement dollars can offset anticipated shortfalls in federal SUD funding.
  ○ Nationally, the anticipated opioid settlement funds cover only 50% of what has been provided by federal funding from SAMHSA.
  ○ Findings indicate that opioid settlement funds could replace only a limited share of overall SAMHSA SUD state funding, suggesting that substantial gaps in treatment and prevention financing would remain if larger‐scale federal budget cuts were enacted.

## 1 Introduction

The Substance Abuse and Mental Health Services Administration (SAMHSA) is one of the largest sources of federal funding for substance use disorder (SUD) treatment and prevention services in the United States [1]. However, SAMHSA is facing substantial budgetary reductions as of 2025. The Trump Administration's proposed budget includes a $1 billion cut to SAMHSA's operating funds [2], which represents approximately 13% of SAMHSA's $7.5 billion budget in 2024 [2]. Reductions target grants for SUD treatment, prevention, and related support services. Additionally, the Administration proposed consolidating health agencies under the proposed Administration for a Healthy America (AHA). The consolidation would restructure block grants supporting SUD services. Both the federal cuts and restructuring plans place funding for SUD treatment and prevention services in jeopardy [3].

In light of these proposed changes, attention has shifted to the distribution of funds from national settlement agreements with opioid distributors, manufacturers, and retailers. These agreements require states to spend at least 85% of the settlement funds on opioid remediation efforts, which include expansion of access to medications for opioid use disorder (MOUD), distribution of the overdose reversal drugs such as naloxone, and improvements in care coordination services [4]. Settlement funds are divided between state and local governments, although the distribution differs across states [5]. Notably, there are no federal enforcement mechanisms to ensure state compliance, leaving room for misuse. For example, emerging evidence suggests that some jurisdictions have allocated these resources toward policing‐related activities, rather than activities recommended by experts [6]. Amid anticipated federal budget reductions in SUD services, some states are considering opioid settlement funds as a replacement for federal funds [7].

As federal support for financing SUD services recedes, state and local governments may rely on settlement funds to sustain SUD services. This is concerning, given the ongoing severity of the overdose crisis—with more than 80,000 overdose deaths reported in 2024 [8] and persistent gaps in access to SUD treatment across the United States [9]. Enhanced investment in overdose prevention and treatment infrastructure is needed. Experts believe that settlement funds are not sufficient to offset the gap left by federal disinvestments [7]. In this study, we analyze SAMHSA federal funding awards alongside projected state‐level opioid settlement distributions to evaluate the potential of opioid settlement dollars disbursed to state and local governments to replace federal SUD funding. Furthermore, we examine whether states with higher overdose death rates would have their SAMHSA SUD treatment and prevention service funding replaced at a higher level by the opioid settlement funds.

## 2 Methods

### 2.1 Data Source and Measures

We obtained data on estimated opioid settlement distributions from KFF Health News (KFF) [10]. Data were collected and coded by KFF in partnerships with researchers from Shatterproof and Johns Hopkins University, and the methodology is described elsewhere [11]. Using KFF's internal 2022–2038 data on payouts and projections, we created five separate measures for each state: (1) the average annual estimated per capita disbursements from 2025 to 2038, which characterizes future disbursements; recognizing that some states may not yet have spent funds disbursed in earlier years, we calculated (2) the average of both paid and estimated annual disbursements from 2022 to 2038; given that variation in the structure and timing of settlement payments may cause differences in payouts across years, we extracted (3) the highest and (4) lowest annual disbursements for each state between 2025 and 2038; to enable a more direct comparison of funds over which state governments have spending authority, we (5) limited both the settlement and SAMHSA funding measures to amounts allocated specifically to state governments. This last measure used only SAMHSA grants awarded directly to state governments and applied the percentage of settlement funds designated for state‐level use as opposed to local use [12]. Ten states were excluded from this analysis because they did not receive a distinct state share of settlement funds (i.e., funds were awarded to state opioid abatement councils or similar entities rather than state governments). West Virginia was excluded from all analyses due to missing information in the KFF datafile. Next, we converted each of these values to a per capita measure using the estimated number of individuals with an SUD in each state in 2022 from the National Survey of Drug Use and Health (NSDUH)—the most recent year for which estimates were available [13]. We calculated per capita measures to improve interpretability by standardizing the scale of amounts across states. We used the estimated number of individuals with an SUD as the denominator as this approach aligns with the objectives of opioid remediation, to fund activities aimed at treating and reducing harms associated with substance use, and reflects how settlement funds are allocated. Allocation is determined using a formula that weighs three factors related to opioid use: opioid‐related deaths, prevalence of opioid use disorder (OUD), and prescription opioid shipment volume [14]. As a sensitivity analysis, we calculated the average annual estimated per capita disbursements from 2025 to 2038 using the total population in each state in 2023 from the American Communities Survey (ACS) as the denominator, the most recent year for which these estimates are available [15]. We conduct this sensitivity analysis because services funded by the settlement dollars and SAMHSA grants may also benefit populations without SUDs.

We compiled data on SAMHSA‐funded SUD awards from two sources: the Tracking Accountability in Government Grants System (TAGGS) and SAMHSA's online grants dashboard. We extracted Notice of Funding Opportunity (NOFO) titles, grant titles, and abstracts from SAMHSA's website [16, 17]. These materials were systematically coded as SUD‐focused via a keyword search. The list of keywords used is provided in Appendix Table 1 in Supporting Information. Three team members independently coded grants to ensure consistency and accuracy. We merged SAMHSA data with TAGGS administrative grant records by matching NOFO numbers across both sources. We also acquired the following variables from the TAGGS data: state, fiscal year, recipient class, and funding award total (in US dollars). We aggregated SAMHSA data to the state‐level, and converted total funding amounts to per capita values based on the estimated total number of individuals in the state with an SUD in the prior year (2022). To assess differences in the replacement of SAMHSA SUD funding by opioid settlement funds, we obtained 2023 state‐level overdose death counts from the Centers for Disease Control and Prevention [18]. We assigned states to quartiles based on overdose death rates per 100,000 population using 2023 state population estimates from the ACS [15].

### 2.2 Statistical Analysis

For our primary analysis, we subtracted the average estimated annual per capita opioid settlement distributions from 2025 to 2038 from the mean per capita amount of federal SAMHSA SUD treatment and prevention service funds in 2024. This quantified the absolute monetary difference between the two funding sources. Next, for each state, we calculated the percentage of federal SAMHSA SUD treatment and prevention service funds that could be replaced by future opioid settlement funds. For example: if Kentucky received $90 per capita from federal SAMHSA funds for SUD treatment and prevention services in 2024, and Kentucky anticipated receiving $45 per capita in settlement funding for SUDs in 2025, this would represent a 50% offset.

We calculated absolute differences and percentages nationally, by state, and stratified by states' overdose death rate quartile. We also conducted a one‐way analysis of variance (ANOVA) to test for differences in means across quartiles. We conducted a sensitivity analysis excluding Oregon, which received less SAMHSA SUD funding than other states, from national calculations.

Sensitivity analyses also explored the percentage of SAMHSA federal SAMHSA SUD treatment and prevention services funds potentially filled by opioid settlement funds using the total disbursements from 2022 to 2038, the highest and lowest annual payments, and funds allocated specifically to state governments.

Analyses were performed using Stata SE 18. This study follows the STROBE reporting guidelines for cross‐sectional studies and was considered exempt from review by the Rutgers University Institutional Review Board as the study does not involve human subjects.

## 3 Results

Descriptive statistics are presented in Table 1. The mean overdose mortality rate across all states in 2023 was 33.03 deaths per 100,000 population (SD: 14.15), ranging from a mean of 17.51 (SD: 4.59) in Quartile 1 of states to 50.72 (SD: 14.33) in Quartile 4. In 2024, the mean SAMHSA SUD award per capita across all states was $97.67 (SD: $55.23), with higher levels observed among states in Quartile 4 of overdose deaths ($121.69; SD: $91.28) compared to those in the lower quartiles (Quartile 1—$90.74, SD: $30.49; Quartile 2—$84.69, SD: $34.74). Mean estimated future opioid settlement disbursements per capita (2025–2038) were relatively similar across groups, averaging $40.83 (SD: $10.00) overall. The differences in mean overdose death rate and SAMHSA SUD funding across quartiles were statistically significant (*p* < 0.01).

**TABLE 1:** State‐level descriptive statistics, overall and by quartiles of 2023 overdose death rates (*n* = 50).

Variable	Total (*n* = 50)	States in Quartile 1 of overdose deaths (*n* = 13)	States in Quartile 2 of overdose deaths (*n* = 12)	States in Quartile 3 of overdose deaths (*n* = 13)	States in Quartile 4 of overdose deaths (*n* = 12)
Overdose deaths per 100,000 population (SD)^a^ ^,^ **	33.03 (14.15)	17.61 (4.59)	28.96 (3.00)	35.88 (2.87)	50.72 (14.22)
Mean SAMHSA SUD award totals per capita in 2024 (SD)^b^ ^,^ **	97.67 (55.23)	90.74 (30.49)	84.69 (34.74)	94.41 (44.54)	121.69 (91.28)
Mean estimated future settlement disbursements per capita (SD)^b^	40.83 (10.00)	39.55 (10.95)	40.66 (9.41)	41.23 (6.91)	41.95 (13.06)
Difference between mean SAMHSA SUD award totals (2024) and mean future settlement disbursements (SD)**	56.83 (53.76)	51.18 (30.32)	44.03 (29.41)	53.17 (44.07)	79.73 (89.90)
Percentage of mean SAMHSA SUD award totals (2024) filled by mean future settlement disbursements (SD)**	51.16 (28.46)	46.73 (14.84)	51.34 (12.52)	49.75 (16.71)	57.32 (53.29)

^a^ Calculated using 2023 state population totals and overdose death rates.

^b^ Per capita by estimated number of individuals within each state with an SUD.

** *p* < 0.01 in one‐way ANOVA.

**FIGURE 1:**
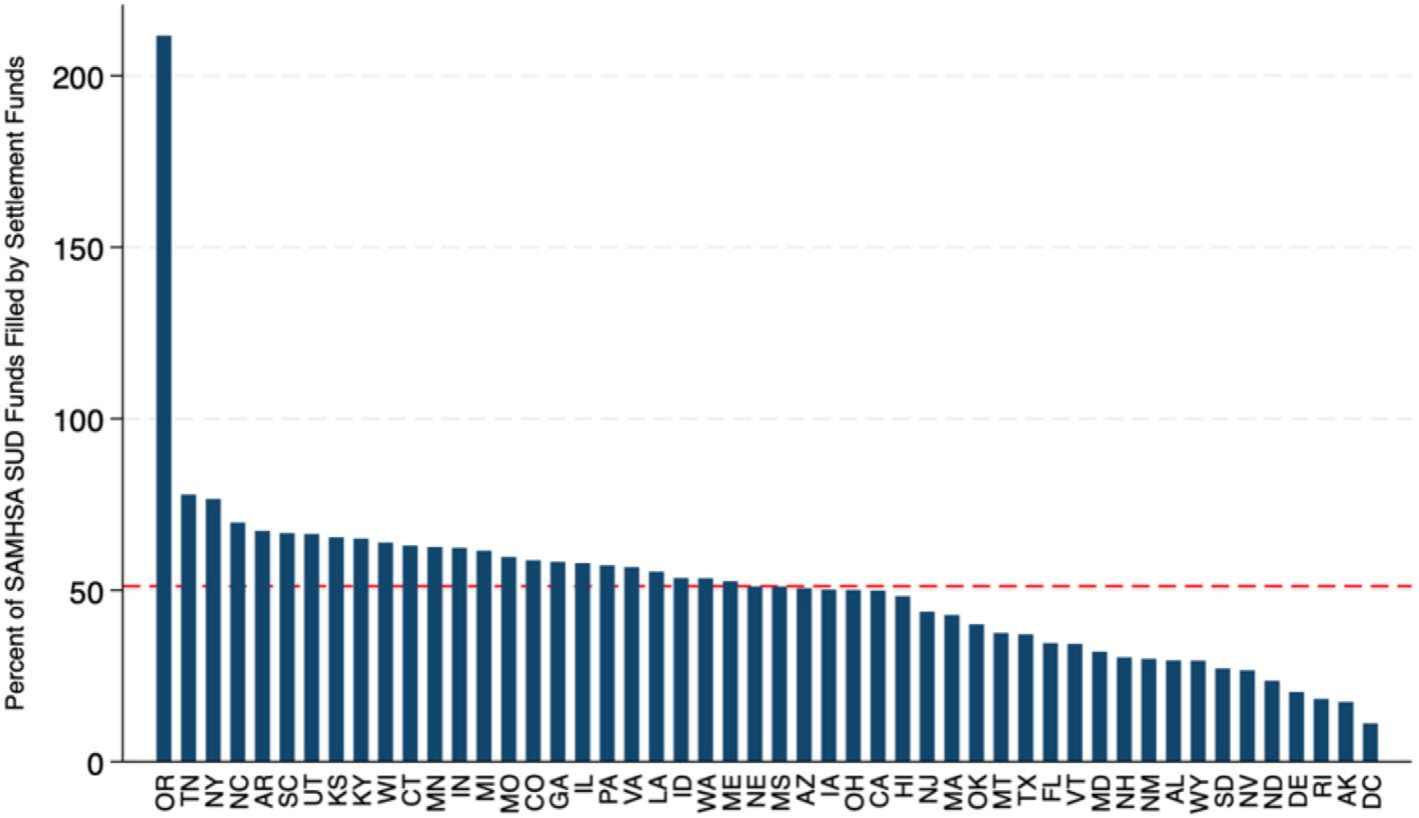
Average of estimated annual settlement funds per capita (2025–2038) as a percent of 2024 SAMHSA SUD per capita funding. Red dashed line represents mean value (51.16).

Figure 1 displays the percentage of per capita SAMHSA SUD funding that we anticipate could be offset by future opioid settlement funds for each state based on current projections. Across states, the mean percentage of SAMHSA SUD funding that could be potentially offset by opioid settlement funds was 51.16% (SD: 28.46%). Oregon was the only state where estimated opioid settlement funds exceeded SAMHSA SUD funds, with the projected offset totaling 211.71%. Among all other states, the highest offset percentages were observed in Tennessee (77.86%), New York (76.54%), and North Carolina (69.69%). In contrast, the lowest replacement percentages were found in the District of Columbia (11.08%), Alaska (17.33%), and Rhode Island (18.21%). When excluding Oregon from the analysis, the mean percentage of SAMHSA SUD funding potentially offset by settlement funds was 47.89% (SD: 16.70).

Appendix Figures 1–4 in Supporting Information present the results of our sensitivity analyses. When incorporating both paid and estimated disbursements from 2022 to 2038, opioid settlement funds were estimated to offset an average of 58.91% of SAMHSA SUD funding (SD: 33.77). Using the highest estimated annual disbursement for each state, opioid settlement funds covered an average of 72.85% (SD: 39.22), whereas using the lowest estimated opioid settlement funds disbursement, the average share covered dropped to 25.67% (SD: 14.62). Finally, when limiting both funding streams to amounts allocated specifically to state governments, the estimated share of SAMHSA SUD funding offset by opioid settlement funds was 20.29% (SD: 12.72). Across states, 87.96% (SD: 14.61) of SAMHSA funds were awarded to state governments.

Using total state population to calculate per capita settlement and SAMHSA SUD funding, opioid settlement funds on average replaced 155.4% (SD: 94.9) of SAMHSA funding, and 14 states received less than full (100%) replacement (Appendix Figure 5 in Supporting Information).

**FIGURE 2:**
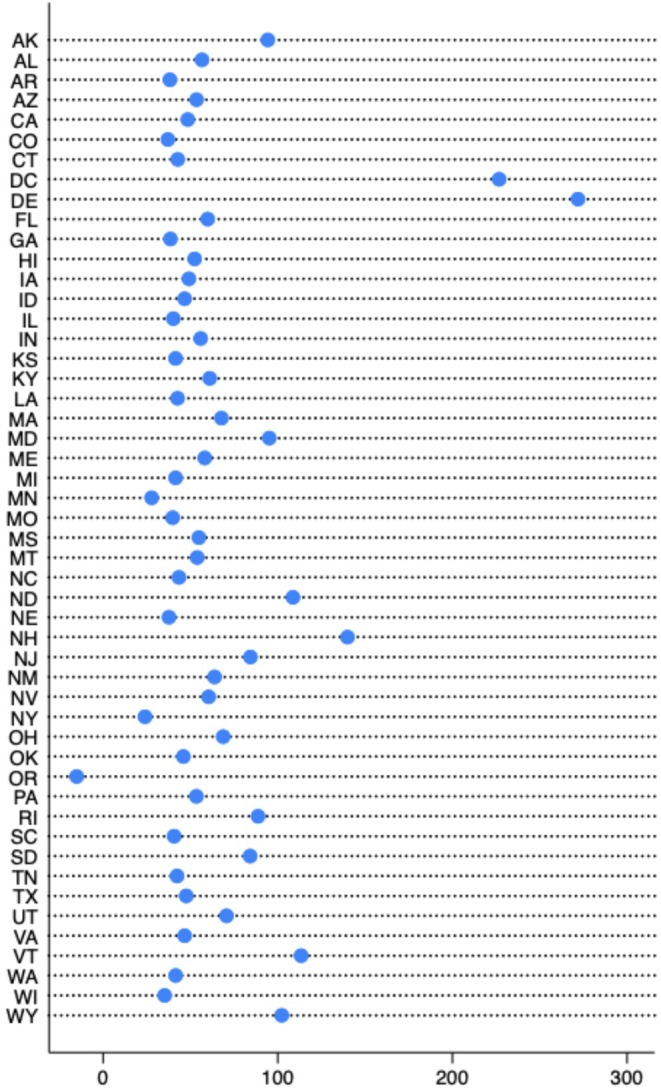
Difference in per capita SAMHSA SUD Funds (2024) versus average of estimated annual opioid settlement funds by state (2025–2038).

Figure 2 reports the difference between the dollar amount of SAMHSA SUD treatment and prevention services funds per capita and anticipated opioid settlement disbursements per capita. Across all states, the mean difference was $56.83 (SD: 53.76).

## 4 Discussion

This study used data on federal SAMHSA SUD grants and estimated future opioid settlement distributions to determine whether opioid settlement funds would be a sufficient offset for all state SAMHSA‐funded SUD services. We found that in most states, the anticipated opioid settlement funds cover about half of what is provided by SAMHSA SUD funds. Further, the difference between SAMHSA SUD funds and opioid settlement disbursements was largest in states with the highest overdose death rates, highlighting potential vulnerabilities in the areas most impacted by the opioid crisis.

Indeed, our findings show that opioid settlement funds could not fully replace existing SAMHSA SUD funding for the populations most affected by substance use. When calculated per individual with an SUD, nearly all states receive less than 100% of SAMHSA funding, revealing a gap in resources relative to treatment need. Oregon is a notable exception, where settlement disbursements appear to exceed SAMHSA funding; this is primarily due to the relatively limited federal funding the state has received historically, so even moderate settlement amounts result in a high ratio. While the drivers of Oregon's comparatively low SAMHSA SUD funding are not fully understood, prior work has documented lower levels of federal SUD funding in Oregon, underinvestment in Oregon's behavioral health system, high rates of mental and SUDs, and high rates of unmet need for SUD treatment [19, 20], suggesting limited capacity and infrastructure.

When per capita values are instead calculated using the total state population in a sensitivity analysis, settlement funds appear to exceed SAMHSA funding in more states. This difference arises because dividing by the much larger total population reduces both per capita values, but not by the same amount, which changes their relative size and makes the settlement‐to‐SAMHSA ratio appear larger. Since both opioid settlement payouts and SAMHSA SUD grants target individuals with SUDs, we find that using a denominator of persons with an SUD is a more accurate per capita measure of the funding intended for this population.

Furthermore, our analysis focuses narrowly on SAMHSA SUD grants, so it likely underestimates the broader funding shortfall that will result when concurrent Medicaid and other health related cuts are considered. For example, the most recent budget resolution specifies cuts to Medicaid of over $880 billion over the next 10 years [2]. Concurrently, the recently passed 2025 Budget Reconciliation Act, known as the One Big Beautiful Bill Act, includes several provisions that impact Medicaid coverage, including additional eligibility determinations and work requirements [21, 22]. Given that Medicaid provided health insurance coverage to nearly half of all adults with OUD in 2023 [23], with rates even higher in states that expanded Medicaid under the Affordable Care Act, these changes would contribute to higher rates of uninsurance among individuals with OUD, reduced access to treatment services, and higher amounts of uncompensated care, leading to increased costs to states [21].

Our findings should be interpreted in light of limitations. First, the data on anticipated opioid settlement disbursements were drawn from KFF, which captures most but not all settlement agreements nationwide. As such, our analysis may underestimate the potential funding replacement. Second, while we conducted a sensitivity analysis on SAMHSA SUD funds earmarked specifically for state governments, SAMHSA SUD dollars also flow to local entities, and there is likewise a local share of settlement funds. Improved reporting and transparency of local opioid settlement spending will be critical to developing a more complete understanding of how these dollars are distributed and used. Third, we obtained estimates of the total number of individuals per state with an SUD from the NSDUH, which likely underestimates the prevalence of SUDs in the United States. Fourth, estimates of budget shortfalls are based on applying funds to the full SAMHSA SUD budget within states and not only to the proposed cuts. Last, we compared opioid settlement funds, which are primarily designated to address OUD, to SAMHSA funding that was intended for SUDs (excluding alcohol and tobacco) but not exclusive to opioids, potentially creating an imprecise comparison between OUD‐specific and broader drug‐related SUD funding streams.

Our findings reveal a substantial difference between current federal funding toward SUD and what future opioid settlement funds could offset. While some states may be tempted to use opioid settlement funds to address broader budget shortfalls, experts caution that such use is not only likely to be insufficient but may also fall outside the intended scope of opioid remediation.

## Funding

Jonathan Cantor and Ryan McBain are supported by funding from the National Institutes of Health (NIH) (1R01MH135034‐01A1).

## Conflicts of Interest

The authors declare no conflicts of interest.

## Supporting information


**Apendix Table 1.** Keywords used to code SAMHSA Notice of Funding Opportunity (NOFO) abstracts for an SUD focus.
**Appendix Figure 1**. Average of paid and estimated settlement funds per capita (2022–2038) as a percent of 2024 SAMHSA SUD per capita funds.
**Appendix Figure 2**. Highest single‐year estimated settlement funds per capita (2025–2038), as a percent of 2024 SAMHSA SUD per capita funds.
**Appendix Figure 3**. Lowest single‐year estimated settlement funds per capita (2025–2038), as a percent of 2024 SAMHSA SUD per capita funds.
**Appendix Figure 4**. Average of estimated annual settlement funds per capita allocated to state governments (2025–2038) as a percent of 2024 SAMHSA SUD per capita funding awarded to state governments.
**Appendix Figure 5**. Average of estimated annual settlement funds per capita by total state population (2025–2038) as a percent of 2024 SAMHSA SUD per capita funding.

## Data Availability

The data that support the findings of this study are available from publicly available data sources. These data were derived from the following resources available in the public domain: KFF Health News Opioid Settlements Payouts, https://kffhealthnews.org/news/article/opioid‐settlements‐payouts‐overdoses‐addiction‐database/; SAMHSA Grants Dashboard, https://www.samhsa.gov/grants/grants‐dashboard; Tracking Accountability in Government Grants System Database, https://taggs.hhs.gov/; CDC restricted access mortality files (must apply for access).
