# Supplementary material for: Examining the Potential Role of Opioid Settlement Funds in the Face of Impending Federal Budget Reductions for Substance Use Disorders
Source: Health Serv Res. 2026 Feb 24;61(2):e70094. doi: 10.1111/1475-6773.70094 (PMC12932015; doi:10.1111/1475-6773.70094)
Supplement: Supplementary file 1 — Apendix Table 1. Keywords used to code SAMHSA Notice of Funding Opportunity (NOFO) abstracts for an SUD focus. Appendix Figure 1. Average of paid and estimated settlement funds per capita (2022–2038) as a percent of 2024 SAMHSA SUD per capita funds. Appendix Figure 2. Highest single‐year estimated settlement funds per capita (2025–2038), as a percent of 2024 SAMHSA SUD per capita funds. Appendix Figure 3. Lowest single‐year estimated settlement funds per capita (2025–2038), as a percent of 2024 SAMHSA SUD per capita funds. Appendix Figure 4. Average of estimated annual settlement funds per capita allocated to state governments (2025–2038) as a percent of 2024 SAMHSA SUD per capita funding awarded to state governments. Appendix Figure 5. Average of estimated annual settlement funds per capita by total state population (2025–2038) as a percent of 2024 SAMHSA SUD per capita funding. [file HESR-61-0-s001.docx]

**Appendix Table 1.** Keywords used to code SAMHSA Notice of Funding Opportunity (NOFO) abstracts for an SUD focus

| **Keywords** |
| --- |
| 'opioid', 'substance use', 'addiction', 'drug', 'overdose', 'fentanyl', 'misuse', 'recovery', 'dependence', 'fentanyl', 'narcotic', 'painkiller', 'opiate', 'methadone', 'buprenorphine', 'methamphetamine', 'screening, brief intervention, and referral to treatment', ‘SBIRT’, ‘harm reduction’, ‘naloxone’, ‘narcan’, ‘MOUD’, ‘needle exchange’, ‘syringe’ |

**Appendix Figure 1.** Average of Paid and Estimated Settlement Funds Per Capita (2022–2038) as a Percent of 2024 SAMHSA SUD Per Capita Funds^a^


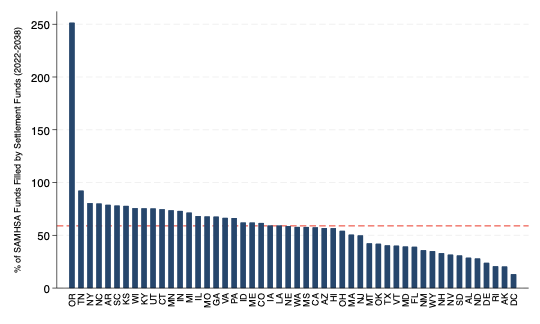


^a^Red dashed line represents mean value (58.91)

**Appendix Figure 2.** Highest Single-Year Estimated Settlement Funds Per Capita (2025–2038), as a Percent of 2024 SAMHSA SUD Per Capita Funds^a^


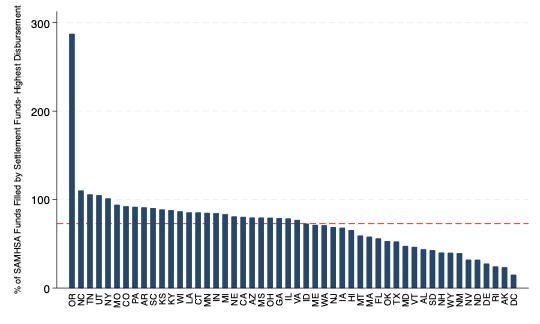


^a^Red dashed line represents mean value (72.85)

**Appendix Figure 3.** Lowest Single-Year Estimated Settlement Funds Per Capita (2025–2038), as a Percent of 2024 SAMHSA SUD Per Capita Funds^a^


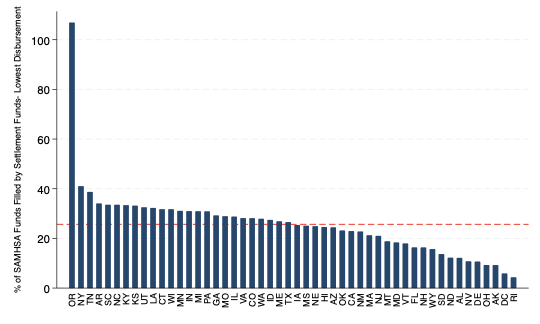


^a^Red dashed line represents mean value (25.67)

**Appendix Figure 4.** Average of Estimated Annual Settlement Funds Per Capita Allocated to State Governments (2025–2038) as a Percent of 2024 SAMHSA SUD Per Capita Funding Awarded to State Governments^a b^


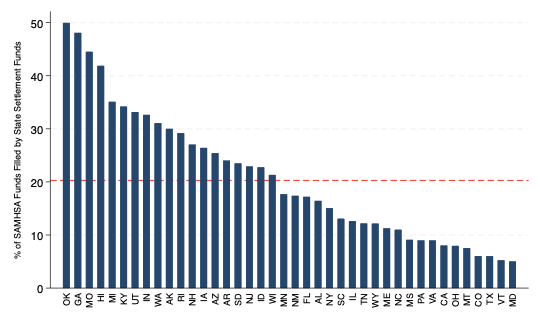


^a^Red dashed line represents mean value (20.29)

^b^States that did not report a percentage of settlement funds allocated to state governments (n=10) were excluded from this analysis

**Appendix Figure 5.** Average of Estimated Annual Settlement Funds Per Capita By Total State Population (2025–2038) as a Percent of 2024 SAMHSA SUD Per Capita Funding ^a^


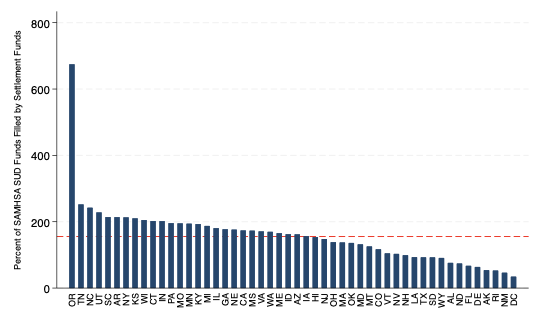


^a^Red dashed line represents mean value (155.40)
